# Antiviral activity of *Hellenia speciosa* (J. Koenig) S.R. Dutta rhizome metabolites against human adenovirus: insights from molecular docking and *in vitro* studies

**DOI:** 10.3389/fphar.2025.1687104

**Published:** 2026-01-13

**Authors:** Sara Alnhhas, Thamer Bouback, Abdulaziz Albeshri, Tasneem Alsahafi, Faisal Al-Sarraj, Faten A. Al-Sulaimany, Mohamed Ali, Yassmin Moatasim, Omnia Kutkat, Mohamed Gaballah, Raied Badierah, Eman O. Taibah, Yaaser Q. Almulaiky, Fawaz Al-Zughaibi, Mohammed Mufrrih, Saleh M. Al-Maaqar, Suzan M. Fathuldeen

**Affiliations:** 1 Department of Biological Sciences, Faculty of Science, King Abdulaziz University, Jeddah, Saudi Arabia; 2 Center of ScientiFIc Excellence for Influenza Viruses, Environmental Research Division, National Research Centre (NRC), Cairo, Egypt; 3 Medical Laboratory, King Abdulaziz University Hospital, King Abdulaziz University, Jeddah, Saudi Arabia; 4 Virology Laboratory, Lab Specialist, King Abdulaziz University Hospital, Jeddah, Saudi Arabia; 5 The Applied College, University of Jeddah, Jeddah, Saudi Arabia; 6 Chemistry Department, Faculty of Applied Science, Taiz University, Taiz, Yemen; 7 Microbiology Department, Pathology and Laboratory Medicine, Prince Mohammed Bin Abdulaziz Hospital tal PMBAH, Medina, Saudi Arabia; 8 Department of Medical Laboratory Sciences, Faculty of Applied Medical Science, King Abdulaziz University, Jeddah, Saudi Arabia; 9 Special Infectious Agents Unit BSL-3, King Fahd Medical Research Center, King Abdulaziz University, Jeddah, Saudi Arabia; 10 Department of Biology, Faculty of Education, Albaydha University, Al-Baydha, Yemen; 11 Laboratory Department, University Medical Services Center, King Abdulaziz University, Jeddah, Saudi Arabia

**Keywords:** human adenoviruses, *Hellenia speciosa* (syn. *Costus speciosus*), antiviral pharmacological effects, molecular docking, GC–MS, drug-likeness, metabolites

## Abstract

**Background:**

Human adenoviruses (HAdVs) cause a wide range of clinical diseases, yet effective antiviral therapies remain limited. Natural products, particularly medicinal plants, offer potential sources of antiviral agents.

**Purpose:**

This study aimed to evaluate the antiviral potential of *Hellenia speciosa* (J.Koenig) S.R.Dutta [syn. *Costus speciosus* (J.Koenig) Sm.; Costaceae] rhizome against HAdVs using both *in vitro* and *in silico* approaches.

**Methods:**

Rhizome extracts were tested on Vero cells to assess cytotoxicity and antiviral activity. GC–MS identified bioactive metabolites, which were screened against adenoviral DNA polymerase using molecular docking.

**Results:**

Docking predicted strong interactions of selected metabolites with the viral polymerase, guiding selection for *in vitro* assays. The methanolic extract exhibited low cytotoxicity and potent antiviral effects, while the ethanolic extract also showed notable activity. Selectivity indices indicated a favorable therapeutic window.

**Conclusion:**

*H. speciosa* rhizome contains bioactivemetabolites with potential anti-adenoviral activity. While preliminary results are promising, further *in vivo* and clinical studies are required to confirm pharmacological relevance.

## Highlights

- *Costus speciosus* extracts exhibited potent antiviral activity against HAdV-C.
- Molecular docking revealed strong interactions with viral DNA polymerase.
- Compounds demonstrated favorable drug-likeness and safety profiles.

## 1 Introduction

**FIGURE 1:**
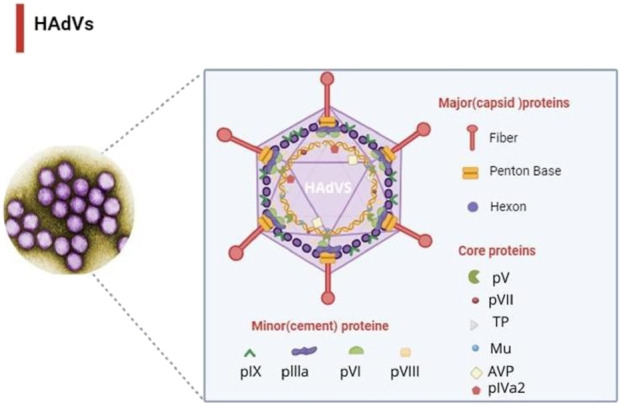
Structural model of the human adenovirus capsid illustrating the major capsid proteins (hexon, penton base, and fiber) and the positions of selected core proteins. This figure was created with https://BioRender.com.

Human adenoviruses (HAdVs) are non-enveloped double-stranded DNA viruses belonging to the family Adenoviridae (Benkő et al., 2022; Norrby et al., 1976). Since their discovery in the 1950s, HAdVs have been recognized as important respiratory pathogens with a wide host range (Rowe et al., 1953; Wigand et al., 1982; Mao et al., 2022; Hllleman and Werner, 1954). The International Committee on Taxonomy of Viruses (ICTV) has identified more than 87 types, grouped into seven species (A–G), with *Mastadenovirus* infecting mammals, including humans (Benkő et al., 2022; Kaján et al., 2020; Hundesa et al., 2006; ROSEN, 1960; Liu et al., 2011). Among these, species C (notably HAdV-C2 and HAdV-C5) are the most prevalent and well characterized, sharing highly similar genomic features and frequently associated with respiratory diseases (Walsh et al., 2011; Lion, 2014). Morphologically, adenoviruses exhibit a conserved icosahedral capsid of approximately 90–100 nm in diameter, composed mainly of hexon, penton base, and fiber proteins (Benkő et al., 2022; Davison et al., 2003) (Figure 1). Epidemiologically, HAdVs are significant contributors to respiratory tract infections, accounting for 5%–10% of pediatric and 1%–7% of adult cases globally (San Martín, 2012; Lynch and Kajon, 2016). Severe infections, including pneumonia, may occur in infants, neonates, and immunocompromised patients, with reported mortality rates of 10%–30% in hospitalized cases and up to 50% in immunocompromised populations (Garvey, 2024). Currently, there are no FDA-approved antiviral therapies for HAdVs, and available agents such as cidofovir and ribavirin demonstrate limited efficacy and notable toxicity (Dodge et al., 2021; CDC, 2025; Salmona et al., 2021). These challenges highlight the urgent need for alternative, safer, and more effective therapeutic strategies.

*Hellenia speciosa* (J. Koenig) S.R. Dutta [syn. *Costus speciosus* (J. Koenig) Sm.; *Costaceae*], commonly known as Malay or crepe ginger, is a perennial medicinal plant of the family Costaceae. It has been traditionally used in Ayurveda, Islamic medicine, and other ethnomedicinal systems for the treatment of fever, inflammation, and metabolic disorders (Maji et al., 2020; Ramya, 2022). Pharmacological studies have reported that *H. speciosa* possesses diverse biological activities, including antidiabetic, antioxidant, anti-inflammatory, antimicrobial, and cytoprotective effects (Pawar and Pawar, 2012; Poulsen et al., 2020). Importantly, several studies have also demonstrated its antiviral potential against herpesviruses (Wang et al., 2011), influenza viruses (Senevirathne et al., 2023), and coronaviruses (Singh et al., 2022). However, its activity against human adenoviruses has not been systematically investigated to date.

Considering the global health burden of adenoviral infections, the limited efficacy of existing antiviral drugs, and the ethnopharmacological relevance of *H. speciosa*, this study aimed to evaluate the anti-adenoviral potential of its rhizome extracts. A combined *in silico* and *in vitro* approach was employed to profile phytochemical constituents via GC–MS, assess molecular interactions with adenoviral DNA polymerase through molecular docking, and experimentally validate antiviral activity using Vero cell culture. This integrative strategy provides the first pharmacological evidence of the therapeutic potential of *H. speciosa* rhizome metabolites against human adenoviruses, bridging ethnopharmacological knowledge with modern antiviral drug discovery.

## 2 Materials and methods

### 2.1 Materials

Chemicals and reagents: All solvents (methanol, ethanol, acetone) and chemicals used in extraction and assays were of analytical grade and purchased from Sigma-Aldrich (United States), unless otherwise specified. Instrumentation: The following instruments were used in this study: rotary evaporator (Buchi, Switzerland), UV–visible spectrophotometer (Shimadzu, Japan), incubator (Thermo Fisher Scientific, United States), biosafety cabinet class II (Esco, Singapore), and inverted phase-contrast microscope (Olympus, Japan).

#### 2.1.1 Plant material

The *H. speciosa* rhizome of *Costus* speciosus was purchased from a verified herbal store in Jeddah, Saudi Arabia. The plant material was taxonomically authenticated by a plant taxonomist at the Department of Biological Sciences, King Abdulaziz University, using morphological characteristics in accordance with standard floras (Chaudhary, 2001). A voucher specimen (accession number: KAU-BIO-2025-001) has been deposited in the departmental herbarium for reference.

#### 2.1.2 Cell line

African Vero cells, derived from the kidney of the African green monkey (*Cercopithecus aethiops*), were used in this study due to their high susceptibility to viral infections and their widespread use in virology research. Cells were obtained from King Abdulaziz University Hospital and cultured in Dulbecco’s Modified Eagle Medium (DMEM) supplemented with 10% fetal bovine serum (FBS) and 1% penicillin-streptomycin. Cell cultures were kept at 37 °C in a humidified incubator with 5% CO_2_.

#### 2.1.3 Virus

Human adenovirus serotype 5 (HAdV-5) used in this study was kindly provided by Prof. Dr. Mohammed Ali (National Research Center, Cairo, Egypt).

### 2.2 Sample preparation

Each *H. speciosa* rhizome was gently scrubbed and rinsed under running tap water for 1 min, followed by thorough washing with sterile distilled water. The cleaned rhizomes were then air-dried in the shade for 1 week, broken into small pieces using a hand mortar, ground to a fine powder with an electric grinder, and sieved through a mesh to remove coarse particles. The resulting powder was stored in a tightly sealed container under dry conditions until further use.

#### 2.2.1 Alcoholic extraction

**FIGURE 2:**
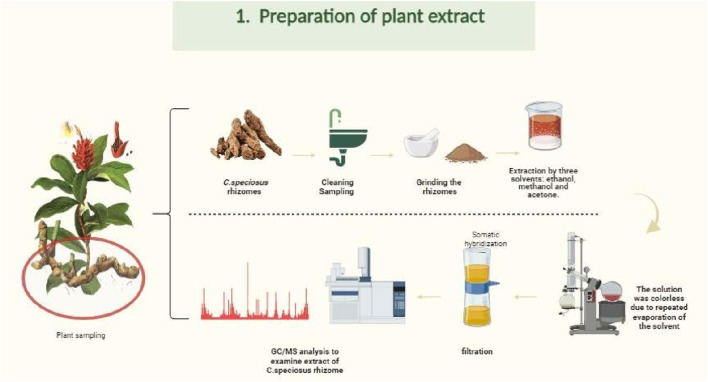
Flowchart of the extraction procedure for *Costus speciosus* rhizomes using methanol, ethanol, and acetone solvents. This figure was created with https://BioRender.com.

For alcoholic extractions, 100 g of dried *H. speciosa* rhizome powder was extracted separately with 400 mL of 80% ethanol, methanol, and acetone using ultrasonic-assisted extraction (UAE). To enhance extraction efficiency, all samples were subjected to ultrasonication at 40 °C for 3 h. Subsequently, the solvent mixtures were concentrated using a rotary evaporator at 70 °C until the solvent became clear and colorless, indicating completion of the extraction cycle (24 h). The resulting extracts were then filtered through Whatman No. 2 filter paper and analyzed by GC–MS (Khan et al., 2016), as shown in Figure 2. Ultrasonic-assisted alcoholic extraction is a widely adopted method for recovering phytochemicals from medicinal plants (Yusoff et al., 2022; Ahmed et al., 2022).

#### 2.2.2 Qualitative detection of alkaloid and non-alkaloid extract

Alkaloids and non-alkaloids were extracted following the method described by Elkady (2013), with slight modifications. Briefly, 100 g of dried rhizome powder was soaked in 200 mL of 80% ethanol for 3 days. The mixture was then filtered and concentrated using a rotary evaporator (Buchi, Switzerland). The resulting crude extract was mixed with 20 mL of glacial acetic acid and 80 mL of distilled water, and the mixture was thoroughly shaken. After filtration through Whatman No. 2 filter paper, an equal volume of chloroform was added, and the solution was agitated for 2 h, resulting in the formation of two distinct layers. The upper layer was discarded, and the pH of the lower aqueous layer was adjusted using 0.1 M hydrochloric acid (HCl). An equal volume of chloroform was added again, followed by shaking for 3 h. This procedure led to the separation of two layers: non-alkaloids were retained in the aqueous layer, while alkaloids were extracted into the chloroform layer. Both layers were immediately freeze-dried and stored for further analysis.

### 2.3 Methods

#### 2.3.1 Gas chromatography-mass spectroscopic analysis of *H. speciosa*

Three solvent extracts (ethanol, methanol, and acetone) of *H. speciosa* rhizome were analyzed using gas chromatography–mass spectrometry (GC-MS) to qualitatively and quantitatively characterize the phytochemical metabolites. The analysis was carried out using an Agilent Technologies GC-Trace Ultra system (Version 5.0) (Sparkman, 2011), equipped with a DB-35-MS capillary column (30 m × 0.25 mm × 0.25 µm film thickness). Helium was used as the carrier gas at a constant flow rate of 1.0 mL/min. The oven temperature was initially set at 60 °C and held for 15 min, then gradually increased to 280 °C and held for 3 min. Compound identification was performed by comparing retention indices and mass spectra with those in the Wiley and NIST libraries. Data analysis and interpretation were conducted in collaboration with the King Fahd Center for Medical Research, King Abdulaziz University, Jeddah, Saudi Arabia.

#### 2.3.2 In silico

##### 2.3.2.1 Sequence alignment

One of the fundamental concepts in bioinformatics is sequence alignment, which has various applications, such as the comparison of DNA or protein sequences to assess their similarity. Techniques commonly employed for this purpose include heuristic algorithms and dynamic programming. In this study, pairwise sequence alignment (Altschul et al., 1990) was used to compare the DNA polymerase proteins of human adenovirus serotype 2 (HAdV-2) and serotype 5 (HAdV-5), as this protein plays a critical role in the viral replication cycle.

The primary amino acid sequences of HAdV-2 (ID: P03261.2) and HAdV-5 (ID: AAW65499.1) were retrieved from the NCBI database in FASTA format. The alignment was performed using the CLUSTALW algorithm (Thompson et al., 1994), a widely adopted method for identifying conserved residues in protein sequences, which can also handle pairwise alignments efficiently. To enhance the visualization of sequence similarity and conserved motifs, the ESPript 3.0 web server was (Robert and Gouet, 2014) employed, generating high-quality graphical representations of the alignment.

##### 2.3.2.2 Preparing protein

Human adenoviruses (HAdVs) are associated with a variety of diseases, particularly respiratory tract infections. Among the different serotypes, types 2 (HAdV-2) and 5 (HAdV-5)—both classified as species C—are the most extensively studied (Chen and Tian, 2018). Due to their high genetic and biological similarity, the findings of studies on HAdV-2 are often applicable to HAdV-5 (Jennings and Parks, 2023). In this study, HAdV-2 DNA polymerase was selected as the target protein for structural and molecular coupling analysis, following a sequence alignment of the HAdV-2 and HAdV-5 polymerases. Homology modeling was performed using the HAdV-2 DNA polymerase sequence obtained from UniProt (ID: P03261) to identify a suitable template (Fatoki, 2023). The modeling was performed using the SWISS-MODEL web server (http://www.swissadme.ch/) (Daina et al., 2017).

The template selected for the modeling process was PDB ID 2pyj.2. C, with a GMQE score of 0.17, a sequence coverage of 32%, and a sequence identity of 19.88%. The final predicted structure was obtained in PDB format. To ensure the reliability of the expected model, its quality was assessed using QMEAN and Ramachandran plot analysis, which confirmed the overall structural plausibility. The modeled protein structure was then prepared for molecular coupling using AutoDock Tools (version 1.5.7) (Trott and Olson, 2010). The active site residues of HAdV DNA polymerase were inferred based on sequence alignment with homologous adenoviral DNA polymerases and available literature, rather than relying solely on the low-identity template (PDB ID: 2pyj, 19.88% sequence identity). We acknowledge that this introduces uncertainty in the exact positioning of these residues; therefore, the docking results are predictive and exploratory, aimed at prioritizing phytocompounds for subsequent *in vitro* validation.

The modeled protein structure was prepared for molecular docking using AutoDock Tools (version 1.5.7) (Daina et al., 2017), where polar hydrogen atoms were added and Gasteiger charges assigned. The active site residues (ASP742, ALA743, LEU772, ARG773, and VAL776) were centered within the docking grid using Discovery Studio, and the final structure was converted to PDBQT format for docking simulations (Jennings and Parks, 2023).

##### 2.3.2.3 Preparing ligands

The crude methanolic extract of *H. speciosa* was first subjected to GC-MS analysis, which revealed 120 physiologically active molecules. Among these, 23 phytocompounds were specifically identified as major metabolites of *H. speciosa*. These metabolites, rather than the crude extract itself, were considered for *in silico* analysis. The corresponding chemical structures of the identified phytocompounds were retrieved from the PubChem database in three-dimensional Structure Data Format (SDF) (Kim et al., 2023; Dassault Systèmes, 2025). The SDF files were converted into PDB format using Discovery Studio. Ligand preparation was carried out in AutoDock Tools (version 1.5.7) (Trott and Olson, 2010) by adding nonpolar hydrogen atoms and computing Gasteiger charges. The finalized ligand structures were saved in PDBQT format and used for molecular docking experiments.

##### 2.3.2.4 Molecular docking simulation

Efficient and accurate molecular docking is critical for virtual screening and the identification of potential lead metabolites (Dias and de Azevedo, 2008). In the present study, docking simulations were carried out using AutoDock Vina (version 1.5.7) (Trott and Olson, 2010). To assess the binding interactions between the individual *H. speciosa* phytocompounds identified by GC-MS and the modeled HAdV DNA polymerase protein, molecular docking simulations were performed. To ensure the reliability of the predicted model, its quality was assessed using QMEAN and Ramachandran plot analysis (Ramachandran et al., 1963). The Ramachandran plot indicated that X% of residues were in the most favored regions, Y% in additionally allowed regions, and Z% in disallowed regions, confirming the overall structural plausibility. These evaluations allowed us to proceed with docking while interpreting the results with caution due to the inherent limitations of the template.

**FIGURE 3:**
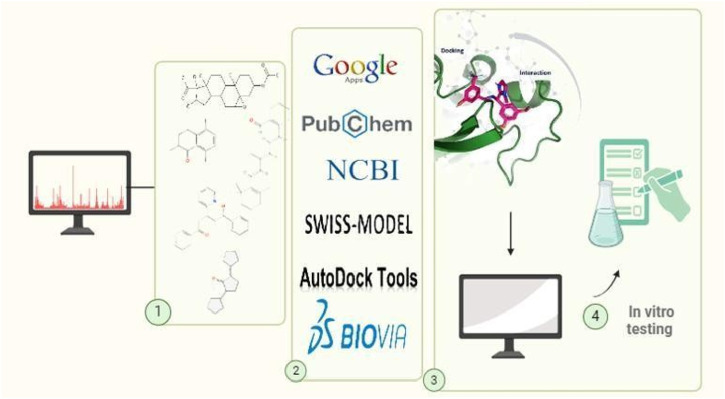
Workflow of the *in silico* molecular docking approach used for screening phytochemicals against adenoviral DNA polymerase. This figure was created with https://BioRender.com.

The docking protocol involved defining a grid box centered on the active site residues of the target protein (dimensions: X = 56, Y = 82, Z = 64). Each ligand was docked independently, and the resulting complexes were evaluated based on their binding affinities (kcal/mol). The top-scoring metabolites were selected for further visualization and detailed structural interaction analysis using BIOVIA Discovery Studio Figure 3. The stability of ligand–protein complexes was further supported by the extensive network of hydrophobic and hydrogen-bond interactions, suggesting energetically favorable conformations. Re-docking of the reference drug (cidofovir) produced an RMSD <2.0 Å, validating grid parameters and scoring reliability. These stability criteria substantiate the predictive robustness of our docking protocol.

It should be emphasized that docking results are predictive in nature and were used to prioritize metabolites for further *in vitro* validation, rather than as direct evidence of pharmacological activity.

##### 2.3.2.5 Protein molecular dynamics simulation

Molecular dynamics (MD) simulations of the protein structure were carried out to evaluate its conformational stability and flexibility. The simulation was performed using the CABSflex2 web server (WAXSiS, 2025), which employs a coarse-grained modeling approach to estimate the residue-level fluctuations of the protein backbone. The default parameters were used, and the Root Mean Square Fluctuation (RMSF) values were calculated for each residue to assess flexibility.

Additionally, the WAXSiS (Small- and Wide-Angle X-ray Scattering) server [http://waxsis.uni-goettingen.de] (WAXSiS, 2025) was used to determine the radius of gyration (Rg) and analyze the compactness of the protein structure in solution. A total of 55 simulation frames were generated, with a total simulation time of 30.5 ps after equilibration. The resulting data were used to evaluate the structural stability and global folding of the protein.

##### 2.3.2.6 Drug likeness analysis and water solubility prediction

The absorption, distribution, metabolism, and excretion (ADME) profile of a compound plays a crucial role in determining its physicochemical properties, including hydrophobicity, lipophilicity, gastrointestinal stability, and blood-brain barrier permeability—factors that influence the drug’s elimination through urine or feces (Yamashita and Hashida, 2004).

In this study, the pharmacokinetic properties of the top ten docked metabolites were evaluated using the SwissADME web server (http://www.swissadme.ch/) (Jennings and Parks, 2023), a widely used platform for early-stage drug discovery and safety assessment. Oral bioavailability was assessed according to Lipinski’s Rule of Five, which considers molecular weight, lipophilicity (log P), the number of hydrogen bond donors, and hydrogen bond acceptors—mainly nitrogen and oxygen atoms.

It is noteworthy that highly lipophilic metabolites may not be suitable for oral administration due to poor gastrointestinal absorption. In such cases, alternative administration routes, such as parenteral (injectable) delivery, may be more effective for faster onset of action.

To analyze drug-likeness and similarity profiles, the Canonical SMILES notation for each selected compound was retrieved and entered into the SwissADME tool web server (http://www.swissadme.ch/) (Jennings and Parks, 2023). The platform provided predictions of physicochemical properties, pharmacokinetics, and drug-likeness scores. Water solubility was assessed using three predictive models integrated within SwissADME: the ESOL model (Daina et al., 2017), the Ali model (Delaney, 2004), and the SILICOS-IT model (Ali et al., 2012).

The ADME and physicochemical properties of these metabolites were compared to the reference anti-adenoviral drug cidofovir, highlighting the relative drug-likeness and therapeutic potential of the phytocompounds.

##### 2.3.2.7 Toxicity risk assessment

Computational methods, particularly *in silico* techniques, have significantly advanced the ability to assess the safety profiles of chemical metabolites. These methods offer efficient, cost-effective, and early-stage evaluation of potential toxicity, which is essential in minimizing risks to human and animal health during drug development (Sepay et al., 2020). In this study, toxicity prediction was performed using the ProTox-II web server, a widely recognized platform for virtual toxicological screening. The tool predicts various toxicity parameters, including the lethal dose 50 (LD50) and classification into toxicity classes, based on the Globally Harmonized System (GHS) for chemical classification and labeling (Opo et al., 2021).

Each compound was assessed by submitting its Canonical SMILES notation, obtained from the PubChem database, to the ProTox-II web server. The platform estimated the median lethal dose (LD50, expressed in mg/kg) and assigned each compound to a corresponding toxicity class based on the Globally Harmonized System (GHS) for chemical classification. This enabled a comparative evaluation of the predicted safety profiles of the selected phytochemical metabolites.

#### 2.3.3 *In vitro* studies

##### 2.3.3.1 Virus propagation

**FIGURE 4:**
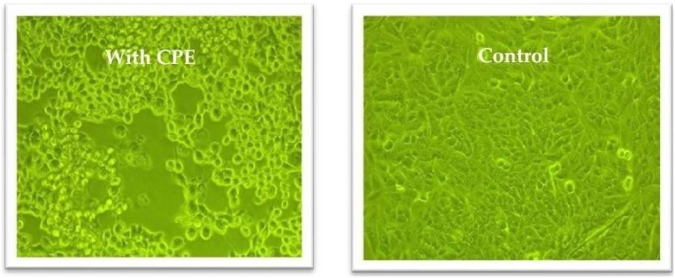
Cytopathic effects in Vero cells following adenovirus infection compared to uninfected controls, observed using an inverted microscope at ×20 magnification.

Vero cells were seeded in T25 flasks and cultured in complete growth medium until reaching approximately 90% confluence. On the following day, the growth medium was carefully removed, and the cell monolayer was rinsed with 1 mL of phosphate-buffered saline (PBS) to eliminate residual serum proteins. Subsequently, the cells were infected with 200 µL of human adenovirus and incubated at 37 °C for at least 45 min to allow viral adsorption. After 15 min of incubation, the flasks were gently agitated to enhance virus-cell contact. Following the adsorption period, 5 mL of infection medium was added to each flask, and the cultures were incubated for 2–3 days at 37 °C in a humidified atmosphere containing 5% CO_2_ Figure 6(1). Infected cells were monitored daily for the appearance of cytopathic effects (CPE), as shown in Figure 4. Once visible CPE was developed, the infected cell cultures were harvested and subjected to centrifugation at 3,000 × g for 15 min at 4 °C to remove cellular debris. The clarified supernatant containing the virus was either used immediately for experiments or aliquoted and stored at −70 °C for future use.

##### 2.3.3.2 Virus titration and median tissue culture infectious dose (TCID50)

Human adenovirus type 5 (HAdV-5), kindly provided by Prof. Dr. Mohammed Ali (National Research Center, Cairo, Egypt) and stored at −70 °C until use, was used to determine the tissue culture infectious dose required to infect 50% of Vero cell monolayers (TCID_50_) using the method described by Reed and Muench (1938) and Banerjee et al. (2018). Crystal violet staining was employed to facilitate microscopic visualization of the cytopathic effects (CPE).

Twenty-four hours before the experiment, 100 µL of a Vero cell suspension containing approximately 1 × 10^3^ cells was seeded into each well of a flat-bottomed 96-well plate. The cells were maintained in Dulbecco’s Modified Eagle Medium (DMEM) supplemented with 10% fetal bovine serum (FBS) and 2% antibiotic/antimycotic solution. The plates were incubated at 37 °C with 5% CO_2_ until the cells reached post-confluence.

Serial tenfold dilutions of the viral stock were prepared using infection medium consisting of DMEM supplemented with 4% bovine serum albumin (BSA) and 2% antibiotics/antimycotics. Ten sterile 1.5 mL microcentrifuge tubes were prepared, each containing 450 µL of infection medium. To initiate the dilution series, 50 µL of the virus stock was added to the first tube to create a 10^−1^ dilution. After thorough mixing, 50 µL was transferred from tube to tube to complete a dilution series from 10^−1^ to 10^−8^.

**FIGURE 5:**
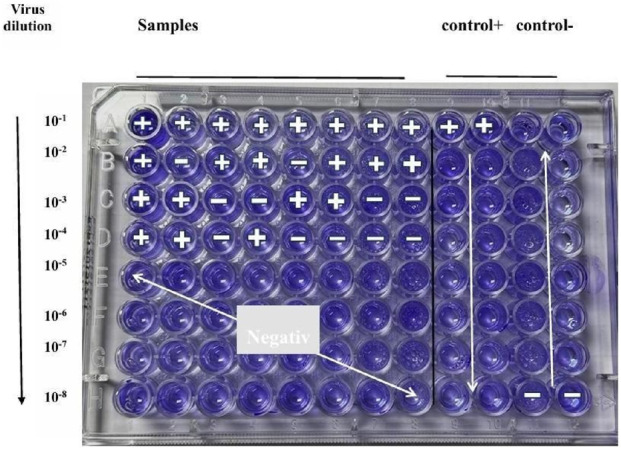
TCID_50_ assay for determining adenovirus titer in Vero cells. Cytopathic effects (CPE) were monitored daily, and virus titer was calculated using the Reed and Muench method (Banerjee et al., 2018).

**FIGURE 6:**
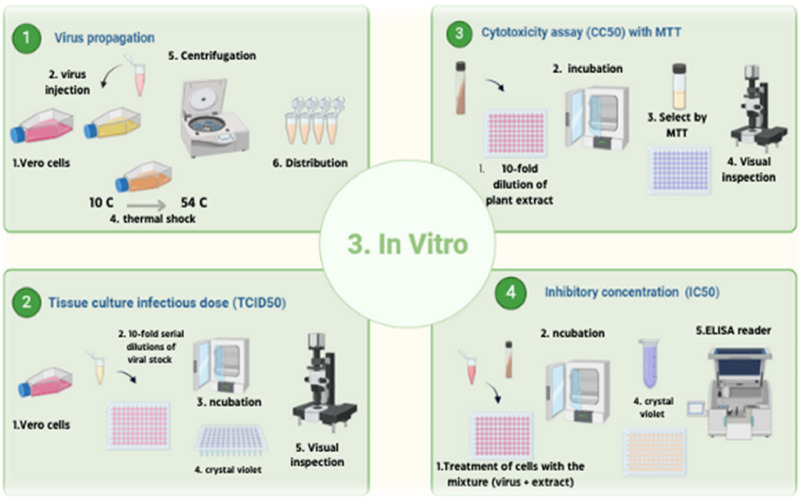
Experimental design overview summarizing phytochemical profiling, molecular docking, and *in vitro* antiviral assays. This figure was created with https://BioRender.com.

Next, 90 µL of each dilution was added to one row (10 wells) of the 96-well plate, with 10 replicates per dilution. The last two wells of each row served as controls: the 11th and 12th wells received 100 µL of virus-free DMEM to serve as negative (cell-only) controls, while undiluted viruses served as a positive control. Plates were incubated at 37 °C in a 5% CO_2_ humidified incubator for 72 h, with daily monitoring for CPE using an inverted microscope see Figure 5. Following incubation, the culture medium was removed, and 50 µL of 0.1% crystal violet solution was added to each well. The plates were incubated for 30 min at 37 °C to allow staining. After staining, the wells were visually inspected for cytopathic effects (CPE). The number of wells showing CPE at each dilution was recorded and analyzed using the Reed-Muench method to calculate the TCID50 value Figure 6(2). The tissue culture infectious dose required to infect 50% of Vero cell monolayers (TCID50) was calculated using the Reed–Muench method. First, cytopathic effects (CPE) were assessed by visual inspection under an inverted microscope, and the number of positive and negative wells was recorded for each virus dilution, excluding control wells. Next, cumulative positive wells were counted starting from the highest dilution (most dilute) to the lowest, while cumulative negative wells were counted in reverse order. The infection rate at each dilution was then calculated using the formula (Reed and Muench, 1938).

Infection Rate = A/(A + B), where *A* represents the cumulative number of positive wells and *B* represents the cumulative number of negative wells. The dilution at which the infection rate approached 50% was considered the endpoint. In this study, a 53% infection rate was observed at the 10^*−*3^ dilution, which was selected as the point surrounding the 50% endpoint. The proportional distance (PD) was then calculated using the formula: The proportionate distance (PD) was calculated as follows (Banerjee et al., 2018):

$$\text{PD}=\frac{53-50}{53-21}+\frac{3}{32}=0.09$$

Finally, the TCID50 value was determined using the Reed–Muench equation: TCID50 = log dilution above 50% + (PD × log dilution factor), resulting in a final value of (Banerjee et al., 2018):

$$\text{TCID}50=-3+\left(-0.09\times 1\right)=-3.09.$$

This value corresponds to the viral dilution that would infect 50% of the Vero cell population.

##### 2.3.3.3 Cytotoxicity assay (CC50) with MTT

To evaluate the cytotoxicity of selected metabolites, the MTT assay was performed using a slightly modified protocol based on the CyQUANT™ MTT Cell Proliferation Assay Kit (V-13154). The aim was to determine the concentration of the compound that induces 50% toxicity in Vero cells. A total of 100 µL of Vero cell suspension was seeded into each well of a 96-well plate and incubated in complete growth medium at 37 °C in a humidified atmosphere with 5% CO_2_ for 24 h, until the cells reached approximately 80%–90% confluence.

Stock solutions of the test metabolites were freshly prepared in 10% DMSO diluted in dis-tilled water (ddH2O) and used on the same day. To prepare for working dilutions, the metabolites were serially diluted in DMEM in a separate cell-free 96-well plate, with four replicates per dilution. For the initial dilution, 180 µL of infection medium was added to wells in row 1, and 100 µL to wells in rows 2 through 12. A 1:10 dilution was prepared by adding 20 µL of the stock extract into row 1 (wells A1 to H1). Then, 2-fold serial dilutions were performed across columns 2–10 by transferring 100 µL from one well to the next, discarding the final 100 µL to maintain volume consistency.

After 24 h of incubation, the growth medium in the cell-seeded plate was aspirated, and the cells were washed twice with sterile phosphate-buffered saline (PBS). Then, 100 µL of each diluted compound was added to the wells containing the Vero cells. Plates were incubated for 48 h at 37 °C with 5% CO_2_. After the incubation period, the medium was removed and replaced with 20 µL of MTT solution diluted in PBS. Plates were incubated for 3–4 h in the dark to allow the formation of formazan crystals. Because MTT is light-sensitive, the plates were properly shielded during incubation.

Following incubation, 50 µL of dimethyl sulfoxide (DMSO) was added to each well to dissolve the formazan crystals, and the plate was incubated for an additional 10 min at room temperature. The MTT assay is based on the enzymatic reduction of the yellow tetrazolium salt MTT into purple formazan crystals by metabolically active cells, thus serving as a direct indicator of cell viability and mitochondrial function. The absorbance of the solubilized formazan was measured using a microplate reader at 540 nm, with 620 nm used as a reference wavelength Figure 6(3).

##### 2.3.3.4 Inhibitory concentration (IC_50_)

The inhibitory concentration required to reduce the viral cytopathic effect by 50% (IC_50_) was determined with slight modifications to previously reported methods (Reed and Muench, 1938; Lei et al., 2021). Vero cells (2.4 × 10^4^ cells/well) were seeded in 96-well plates and incubated overnight at 37 °C in a humidified atmosphere containing 5% CO_2_, allowing the cells to reach approximately 90% confluency.

In a separate 96-well plate (without cells), 180 µL of infection medium was added to the wells in the first row (A1–H1), and 100 µL to the remaining wells (rows 2–10). Rows 11 and 12 received 100 µL each and served as virus and cell controls. To prepare the treatment dilutions, 20 µL of the prepared plant extract stock solution was added to the first row, resulting in a 1:10 dilution. A 2-fold serial dilution was then performed across columns 1 through 10 by transferring 100 µL from one column to the next, discarding the final volume from the last column.

Next, 100 µL of virus suspension containing 100 TCID_50_ was added to each well, except for the virus-free control wells. Additionally, a 10^−3^ dilution of the virus was prepared and applied to wells in rows 1–12, columns E through H, serving as the virus control. Rows 11 and 12 also served as reference controls. The virus-compound mixtures were incubated at 37 °C for 1 h in a humidified atmosphere containing 5% CO_2_ to facilitate interaction.

After incubation, 100 µL of each virus-compound mixture was added to the corresponding wells of a separate 96-well plate containing the pre-incubated Vero cells. The plates were then incubated for 72 h at 37 °C in a humidified atmosphere with 5% CO_2_. Following incubation, cells were fixed with 10% formaldehyde for at least 2 h at room temperature. After fixation, cells were washed and stained with 50 µL of 0.5% crystal violet solution (prepared by dissolving 0.5 g of crystal violet powder in 80 mL distilled water and 20 mL methanol). The stain was allowed to sit at room temperature for 10 min, after which 200 µL of methanol was added to each well. Plates were then left to air-dry completely. Finally, optical density (OD) readings were measured using an ELISA plate reader to assess cell viability and determine the IC_50_ values Figure 6(4).

##### 2.3.3.5 Mechanism of viral inhibition

**FIGURE 7:**
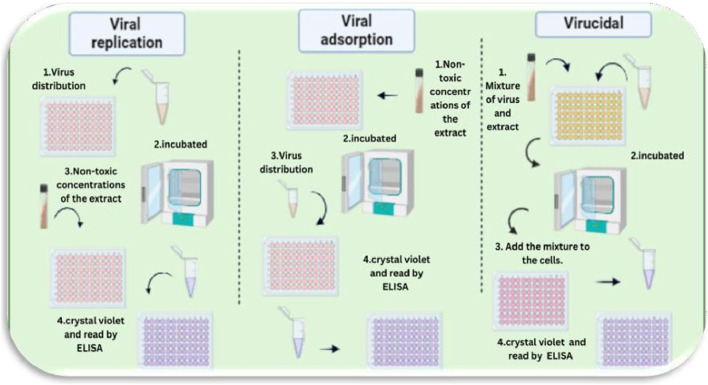
Proposed mechanisms of action of *Costus speciosus* extracts against human adenoviruses (HAdVs). This figure was created with https://BioRender.com.

Viral inhibition refers to the process by which a therapeutic agent or compound disrupts the ability of a virus to infect host cells, replicate, or spread. This inhibition can occur through various mechanisms, including blocking viral entry into host cells, inhibiting viral genome replication, preventing viral protein synthesis, or enhancing the host immune response. A thorough understanding of these mechanisms is crucial for the development and optimization of effective antiviral therapies Figure 7.

###### 2.3.3.5.1 Viral replication

Vero cells were seeded in 96-well plates and cultured for 24 h, or until reaching approximately 90% confluency. The cells were then infected with 100 µL of the virus suspension, prepared at a concentration determined by the TCID_50_ assay, to assess the antiviral activity of *Costus speciosus* extract. Following a 1-h incubation at 37 °C in a 5% CO_2_ atmosphere, the wells were washed three times with 1× PBS. Cells were then treated in replicates with previously determined concentrations of the plant extract (0.5, 1.0, 1.5, and 2.0 μg/mL) and incubated for 72 h at 37 °C in a 5% CO_2_ environment. Control wells (untreated cells and virus-infected controls) were included for comparison. After incubation, the cells were fixed with 10% formaldehyde and left at room temperature for at least 2 h.

Following fixation, the wells were washed and stained with 50 µL of a 0.5% crystal violet solution (prepared by dissolving 0.5 g of crystal violet powder [Sigma-Aldrich] in 80 mL of distilled water and 20 mL of methanol) and incubated at room temperature for 10 min. The plates were then air-dried, and 200 µL of methanol was added to each well for further incubation at room temperature. Optical density (OD) was measured using an ELISA reader. Untreated control cells were considered to represent 100% viability. The average OD values of treated groups (with and without viral infection) were compared to those of the control and virus-only groups to determine the percentage of cytotoxicity and the percentage inhibition of viral replication, respectively. All experiments were performed in triplicate, and data were expressed as mean ± standard error of the mean (SEM).

###### 2.3.3.5.2 Viral adsorption

Five different extracts—ethanol, methanol, acetone, alkaloid, and non-alkaloid—were tested at four concentrations (0.5, 1.0, 1.5, and 2.0 μg/mL), with three replicates for each dilution. The extracts were added to a confluent monolayer of Vero cells cultured in a 96-well tissue culture plate. Treated cells were incubated at 37 °C in a 5% CO_2_ atmosphere for 2 h. After incubation, the wells were washed three times with 1X PBS, and 100 µL of virus (at 100 TCID_50_/well) was added. Virus control wells consisted of infected but untreated cells, while cell control wells contained untreated and uninfected cells. The plates were incubated for 1 h at 37 °C to allow viral adsorption. Following this, the inoculum was removed, and infection media were added before incubating the plates for an additional 72 h under the same conditions.

Post-incubation, cells were fixed with 10% formaldehyde and incubated at room temperature for at least 2 h. The wells were then washed and stained with 50 µL of 0.5% crystal violet solution per well, followed by a 10-min incubation at room temperature. After air-drying, 200 µL of methanol was added to each well and incubated again at room temperature. The optical density (OD) was measured using an ELISA reader.

Non-treated, uninfected control cells were considered to represent 100% viability. The average OD values for each dilution (both with and without virus) were compared to the control groups to calculate the percentage of cytotoxicity and the percentage reduction in viral replication, respectively. Results were reported as the mean ± standard error of the mean (SEM) from at least three independent experiments.

###### 2.3.3.5.3 Virucidal

This method was employed to evaluate the ability of the tested plant extracts to neutralize virus particles and prevent their entry into host cells. Initially, 100 µL of virus suspension was mixed with 100 µL of different concentrations of plant extracts in a 96-well plate and incubated for 1 h at 37 °C. After this incubation, the mixture was transferred to a new 96-well plate containing a confluent monolayer of Vero cells and further incubated for 1 h under the same conditions.

Following this step, the inoculum was removed, and the wells were washed with 1X PBS. The cells were then fixed using 10% formaldehyde and incubated at room temperature for at least 2 h. After fixation, the cells were washed and stained with 50 µL of 0.5% crystal violet solution per well (prepared by dissolving 0.5 g of crystal violet powder [Sigma-Aldrich] in 80 mL of distilled water and 20 mL of methanol), and incubated for 10 min at room temperature.

After air-drying, 200 µL of methanol was added to each well, followed by incubation at room temperature. Optical density (OD) was then measured using an ELISA reader. Untreated, uninfected control cells were assigned to a viability value of 100%. The average OD values for each treatment (with and without virus) were compared to those of the control and virus control wells to calculate the percentage of cytotoxicity and the percentage reduction in viral replication. Data were expressed as the mean ± standard error of the mean (SEM) from at least three independent experiments.

## 3 Results

### 3.1 GC/MS profiling of *Costus speciosus* methanol, acetone, and ethanol extracts

**FIGURE 8:**
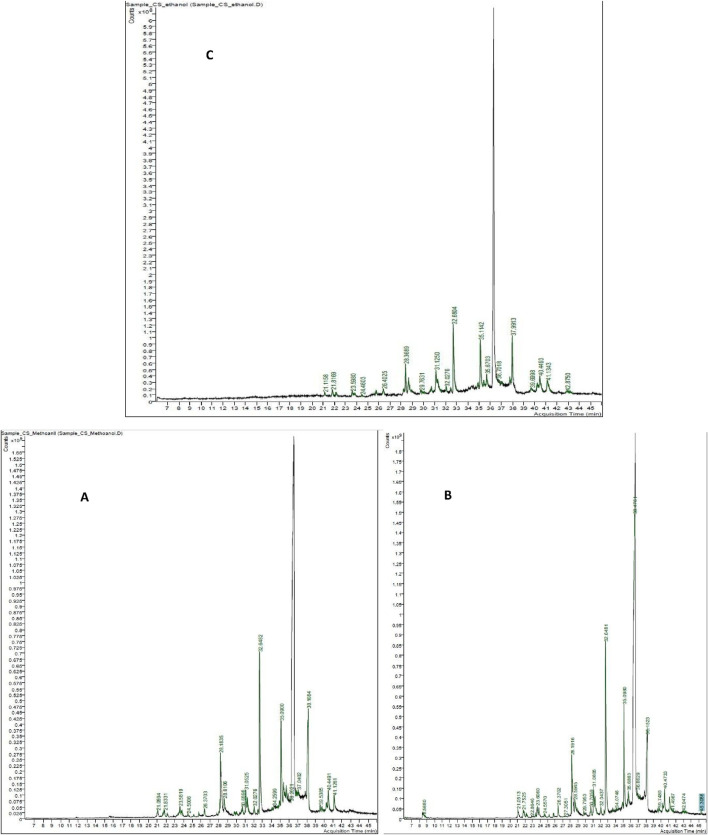
GC–MS chromatograms of *Costus speciosus* rhizome extracts obtained using methanol **(A)**, acetone **(B)**, and ethanol **(C)** as solvents.

The identification of metabolites was carried out by comparing their mass fragmentation patterns with reference spectrum available in the Wiley and NIST database libraries. The GC-MS chemometric analysis is illustrated in Figures 8A–C. The three solvents used for extracting metabolites from *C. speciosus* rhizomes—methanol, ethanol, and acetone—revealed a diverse profile of phytochemicals. These included tocopherols, fatty acids, alkaloids, sesquiterpenes, diterpenes, phenols, and sterols. Table 1 presents the most prominent metabolites identified in the extracts of each solvent, based on their relative abundance.

**TABLE 1:** Phytochemical metabolites of *H. speciosa* isolated from solvents A (Methanol), B (ethanol), and C (acetone).

Solvent	#	Identified name	Rt* (min)	Formula
Solvent A (methanol)				
A	1	2,8-Decadiyne	21.0594	^C^10^H^14
A	2	(+)-3-Carene, 10-(acetylmethyl)-	21.8331	^C^13^H^20^O^
A	3	(3R,4aS,8aS)-8a-Methyl-5-methylene-3-(prop-1-en-2-yl)-1,2,3,4,4a,5,6,8a-octahydronaphthalene	23.5819	^C^15^H^22
A	4	Preg-4-en-3-one, 17.*α*.-hydroxy-17.*β*.-cyano-	24.5006	^C^20^H^27^NO^2
A	5	9,12-Octadecadiynoic acid, methyl ester	26.3703	^C^19^H^30^O^2
A	6	Pyridine, 2-pentyl-	28.1835	^C^10^H^15^N^
A	7	1,8-Cyclopentadecadiyne	28.6106	^C^15^H^22
A	8	Doconexent	30.6898	^C^22^H^32^O^2
A	9	Benzene, 1,1’-[oxybis(methylene)]bis[4-ethyl-	31.0525	^C^18^H^22^O^
A	10	5,8,11,14-Eicosatetraenoic acid, methyl ester, (all-Z)-	32.0276	^C^21^H^34^O^2
A	11	1-(3,3-Dimethyl-but-1-ynyl)-1,2-dimethyl-3-methylenecyclopropane	32.6482	^C^12^H^18
A	12	Azuleno[4,5-b]furan-2(3H)-one, decahydro-3,6,9-tris(methylene)-	34.2599	^C^15^H^18^O^2
A	13	Tricyclo[6.6.0.0(3,6)]tetradeca-1(8),4,11-triene	35.0900	^C^14^H^18
A	14	1(2H)-Naphthalenone, 3,4-dihydro-2,5,8-trimethyl-	36.2021	^C^13^H^16^O^
A	15	Cyclooctene, 1-(9-borabicyclo[3.3.1]nonan-9-yl-2- iodo-, (Z)-	37.0402	^C^16^H^26^BI^
A	16	1,8-Cyclopentadecadiyne	38.1684	^C^15^H^22
A	17	4,7,10,13,16,19-Docosahexaenoic acid, methylester	40.4491	^C^23^H^34^O^2
A	18	Desogestrel	41.1261	^C^22^H^30^O^
Solvent B (ethanol)				
B	1	Doconexent	21.1158	^C^22^H^32^O^2
B	2	cis-5,8,11,14,17-Eicosapentaenoic acid	21.8169	^C^20^H^30^O^2
B	3	Methyl 5,7-hexadecadiynoate	23.5980	^C^17^H^26^O^2
B	4	5,8,11,14-Eicosatetraenoic acid, methyl ester, (all-Z)-	24.4603	^C^21^H^34^O^2
B	5	Acetamide, N-methyl-N-[4-(3-hydroxypyrrolidinyl)-2-butynyl]-	26.4025	^C^11^H^18^N^2^O^2
B	6	3H-Cyclodeca[b]furan-2-one, 4,9-dihydroxy-6-methyl-3,10-dimethylene	29.7631	^C^15^H^20^O^4
B	7	Bicyclo[4.4.0]dec-2-ene-4-ol, 2-methyl-9-(prop-1-en-3-ol-2-yl)-	31.1250	^C^15^H^24^O^2
B	8	O-Arachidonoylglycidol	32.0276	^C^23^H^36^O^3
B	9	(1R,1aR,2aS,5R,6R,6aS,7aS)-…-naphthalen-5-ol	32.6804	^C^15^H^24^O^
B	10	10,13-Octadecadiynoic acid, methyl ester	35.6703	^C^19^H^30^O^2
B	11	Oxazole, 5-ethyl-2-methyl-4-benzoyl-	36.7018	^C^13^H^13^NO^2
B	12	Tricyclo[5.1.0.0(4,6)]octane-3-carbonitrile, 5,5,8,8-tetramethyl-	37.9913	^C^13^H^19^N^
B	13	1-Heptatriacotanol	39.6998	^C^37^H^76^O^
B	14	5,8,11,14-Eicosatetraenoic acid, ethyl ester, (all-Z)-	40.4493	^C^22^H^36O_2_
B	15	Desogestrel	41.1343	^C^22^H^30^O^
B	16	1H-…cyclopropa[e]cyclodecen-11-one…	42.8750	^C^20^H^28^O^6
Solvent C (acetone)				
C	1	dl-Alanyl-dl-*α*-amino-n-butyric acid	8.5680	^C^7^H^14^N^2^O^3
C	2	Bicyclo[4.2.0]oct-1-ene, 7-exo-ethenyl-	21.0513	^C^10^H^14
C	3	Quinoline-2,4(1H,3H)-dione, 3-azido-3-ethyl-1-methyl-	21.7525	^C^12^H^12^N^4^O^2
C	4	Doconexent	22.8646	^C^22^H^32^O^2
C	5	9,12-Octadecadiynoic acid, methyl ester	26.3702	^C^19^H^30^O^2
C	6	O-Arachidonoylglycidol	27.3051	^C^23^H^36^O^3
C	7	Pyridine, 2-pentyl-	28.1916	^C^10^H^15^N^
C	8	Cyclooctene, 4-methylene-6-(1-propenylidene)-	28.5945	^C^12^H^16
C	9	4a,5-Dimethyl-3-(prop-1-en-2-yl)-1-ol	30.7059	^C^15^H^24^O^
C	10	1,8-Cyclotetradecadiyne	31.0605	^C^14^H^20
C	11	5,8,11,14-Eicosatetraenoic acid, methyl ester	32.0437	^C^21^H^34^O^2
C	12	4,7,10,13,16,19-Docosahexaenoic acid, methylester	32.6481	^C^12^H^18
C	13	Tricyclo[6.6.0.0(3,6)]tetradeca-1(8),4,11-triene	35.0980	^C^14^H^18
C	14	Boranamine, 1,1-diethyl-N-phenyl-	35.6863	^C^10^H^16^BN^
C	15	Cyclobutanol, 1-phenyl-	36.4761	^C^10^H^12^O^
C	16	(3R,4aS,8aS)-8a-Methyl…	38.1523	^C^15^H^22
C	17	2-[4-methyl-6-(2,6,6-trimethylcyclohex-1-enyl)…]	39.7480	^C^23^H^32^O^
C	18	Azuleno[4,5-b]furan-2(3H)-one, decahydro-3,6,9-tris(methylene)-17	40.4733	^C^15^H^18^O^2
C	19	1H-…cyclodecen-11-one…	41.4967	^C^20^H^28^O^6
C	20	Ethyl 5,8,11,14,17-icosapentaenoate	42.9474	^C^22^H^34^O^2

### 3.2 In silico

#### 3.2.1 Sequence alignment

**FIGURE 9:**
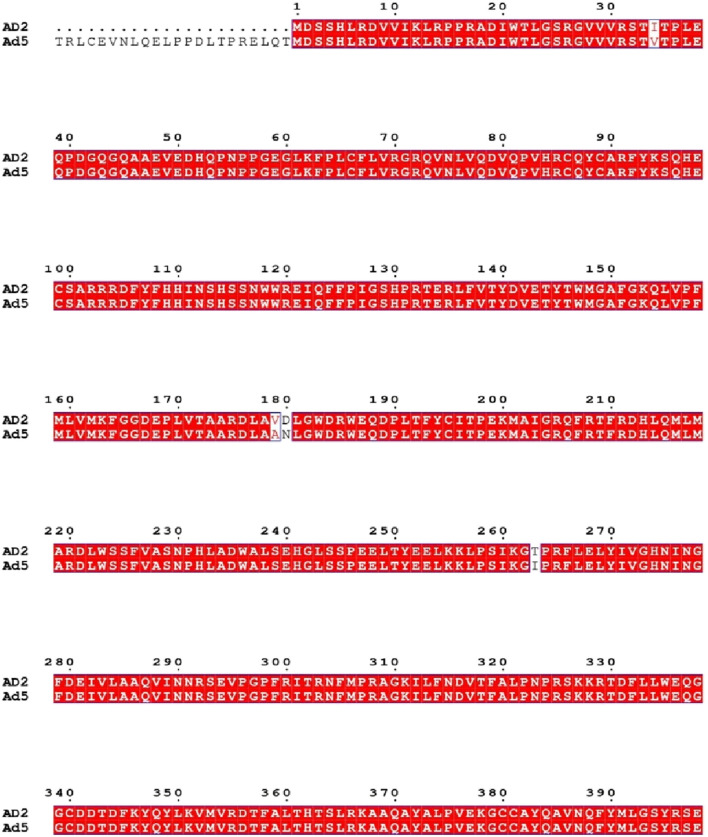
Sequence alignment of adenovirus DNA polymerase proteins from HAdV-2 and HAdV-5. Identical residues are shown in red shading, and differences are highlighted with black boxes (generated using ESPript).

To validate the hypothesis proposed by several studies—that successful antiviral interventions targeting human adenovirus serotype 2 (HAdV-2) may also be effective against serotype 5 (HAdV-5) a comparative genomic analysis was conducted between the two serotypes belonging to HAdV species C. At the genome level, the DNA polymerase proteins of HAdV-2 and HAdV-5 exhibited a remarkably high nucleotide similarity of up to 99.30%. Figure 9 highlights the degree of sequence alignment similarity, with matching regions represented in red.

#### 3.2.2 Molecular docking simulation

This study focused on evaluating the therapeutic potential of metabolites identified through GC-MS analysis for treating HAdV-2 infection. Molecular docking was performed using AutoDock (version 1.5.7) (Trott and Olson, 2010), a widely validated open-source software for structure-based drug discovery. The docking protocol was optimized by re-docking the reference ligand cidofovir into the DNA polymerase active site to validate the accuracy of the docking grid and scoring function. RMSD values <2.0 Å were accepted as validation criteria. AutoDock (version 1.5.7) (Trott and Olson, 2010) was selected due to its accessibility and reproducibility, although we acknowledge that commercial docking platforms such as Schrödinger Glide may offer enhanced accuracy.

A total of 112 metabolites extracted from various parts of *C. speciosus* were screened, and molecular docking (based on binding free energy) was employed to identify the top ten candidate metabolites with strong affinity to the target viral protein. The docking results revealed notable interactions between the selected metabolites—designated CID1 to CID10—and the amino acid residues of the viral protein. These metabolites demonstrated superior binding affinities compared to the reference drug, Cidofovir (CID11). Specifically, CID1 [2-*{*4-Methyl-6-(2,6,6- trimethylcyclohex-1-enyl)hexa-1,3,5-trienyl*}*cyclohex-1-en-1-carboxaldehyde] showed a binding energy of −9.1 kcal/mol with eleven hydrophobic interactions (Pi-Alkyl and Alkyl). CID2 [2-(2-Aminopropanamido)butanoic acid] had a binding energy of −9.0 kcal/mol and formed ten hydrophobic interactions. CID3 [Pregan-20-one, 2-hydroxy-5,6-epoxy-15-methyl-] displayed the strongest interaction at −9.4 kcal/mol, with twelve hydrophobic contacts.

CID4 [Desogestrel] exhibited a binding energy of −8.0 kcal/mol, involving one hydrogen bond and three hydrophobic interactions. CID5 [*2-(2,6-Dimethylphenoxy)-3,6-dimethyl-4- (pentan-3-yloxy)pyridine*] also showed a binding energy of −8.0 kcal/mol and formed six hydrophobic interactions. CID6 [*2,5,8-Trimethyl-1-tetralone*] had a binding energy of −8.4 kcal/mol with one hydrogen bond and three hydrophobic interactions. CID7 [*1,2,3-Triazole- 4-carboxamide, 5-amino-1-phenylsulfonyl-N-(3-methylphenyl)-*] demonstrated a binding energy of −8.7 kcal/mol, forming three hydrogen bonds and two hydrophobic interactions.

CID8[*Cyclopentanone, 2,5-dicyclopentylidene-*] showed a binding energy of −8.6 kcal/mol, forming six hydrogen bonds, six hydrophobic interactions, and one amide bond. CID9[*1,5- Diphenyl-3-(3-cyclopentylpropyl)pentane*] also had a binding energy of −8.6 kcal/mol, with six hydrophobic interactions. CID10[*8a-Methyl-5-methylene-3-([(pyridin-3-ylmethyl)-amino]-methyl)- decahydro-naphtho[2,3-b]furan-2-one*] demonstrated a binding energy of −8.3 kcal/mol, forming eight hydrophobic and three hydrogen bonds. In comparison, the control drug Cidofovir (CID11) exhibited a lower binding energy of −5.6 kcal/mol and formed six conventional hydrogen bonds with residues VAL634, ARG636, TRP806, CYS808, VAL809, and GLU812, along with one unfavorable interaction with ARG811.

Table 2 summarizes the top ten docked metabolites and their interactions with the interface residues of the target protein. The 10 selected phytochemical metabolites established multiple interactions with key amino acid residues, surpassing the reference compound in binding affinity and interaction diversity. Notably, several residues were identified as potential targets involved in the disruption of HAdV-2 host cell recognition. These include PHE630, LEU694, ALA810, LEU843, PHE847, SER663, MET698, PRO699, LEU734, LEU735, PRO736, PHE803, VAL809, ALA848, LEU650, VAL660, ILE664, ARG665, THR778, ALA693, MET503, ASN532, VAL531, PRO497, TYR502, TYR844, and VAL780.

**TABLE 2:** Interaction of *H. speciosa* active metabolites with target proteins through 3D docking.

Target	Ligand (CID)	Binding energy (kcal/mol)	Interacting residues
HAdV DNA pol	CID:1	−9.1	Pi-Alkyl: PHE630, LEU843; Alkyl: LEU650, VAL660, LEU694, MET698, PRO699, PRO736, VAL809, ALA810, PHE847
HAdV DNA pol	CID:2	−9.0	Pi-Alkyl: PHE630, PHE847; Alkyl: LEU650, VAL660, LEU694, MET698, PRO699, VAL809, ALA810, LEU843
HAdV DNA pol	CID:3	−8.0	Pi-Alkyl: TYR502; Alkyl: MET503, VAL531, PRO497; Conventional H-Bond: ASN532
HAdV DNA pol	CID:4	−8.0	Alkyl: ALA693, MET698, PRO699, LEU734, PRO736; Pi-Alkyl: PHE803
HAdV DNA pol	CID:5	−8.4	Alkyl: LEU650, PRO699, ALA848; Conventional H-Bond: TYR844
HAdV DNA pol	CID:6	−8.7	Pi-Sigma: PHE630, LEU694; Pi-Alkyl: ALA810, LEU843; Pi-Pi stacking: PHE847
HAdV DNA pol	CID:7	−8.6	Conventional H-Bond: SER663; Pi-Sigma: MET698; Pi- Alkyl: PRO699, LEU734, PRO736, PHE847; Pi-stacked: LEU735; Pi-Pi stacked: PHE803, PHE847; Alkyl: VAL809
HAdV DNA pol	CID:8	−8.6	Pi-Alkyl: PHE630, PHE847; Alkyl: LEU694, VAL809, ALA810, LEU843
HAdV DNA pol	CID:9	−8.3	Alkyl: LEU650, ILE664, ALA848; Pi-Alkyl: LEU650, VAL660, PRO699, ALA810, LEU843; Pi-Sigma: LEU694; Pi-Pi Stacked: PHE803, PHE847
HAdV DNA pol	CID:10	−8.1	Pi-Alkyl: LEU650, VAL660, PRO699; Conventional H- Bond: THR778, SER779; Carbon-H Bond: THR778; Alkyl: VAL780; Pi-Pi Stacked: PHE803
HAdV DNA pol	CID:11	−5.6	Conventional H-Bond: VAL634, ARG636, CYS808, VAL809, GLU812; Pi-Sigma: VAL634; Pi-Donor: TRP806; Carbon-H Bond: VAL809; Donor-Donor unfavorable: ARG811; positive-positive unfavorable: ARG811

**FIGURE 10:**
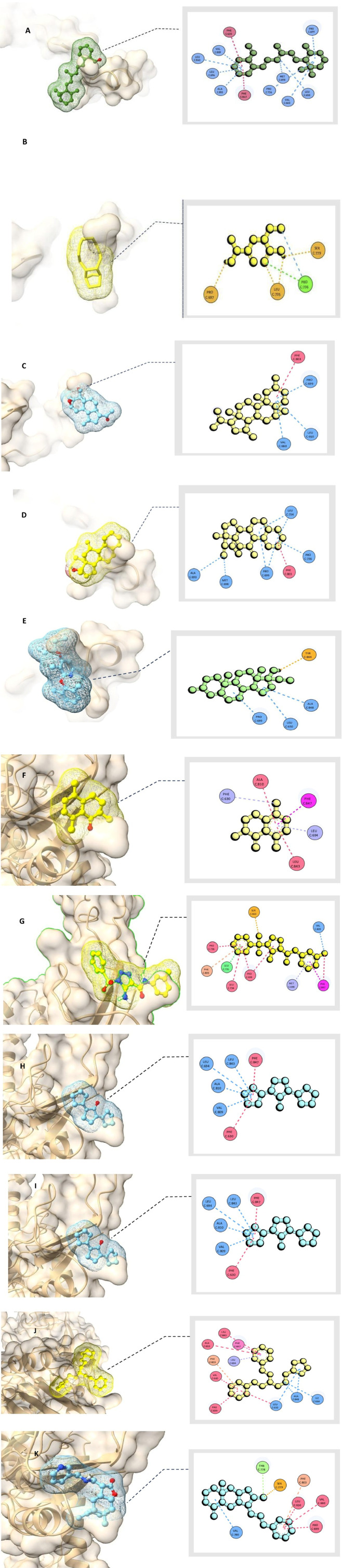
Molecular docking analysis shows binding interactions of natural metabolites with HAdV-2 DNA polymerase (Uniprot ID: P03261) in 2D and 3D visualizations **(A)** [2-*{*4-Methyl-6-(2,6,6- trimethylcyclohex-1-enyl)hexa-1,3,5-trienyl*}*cyclohex-1-en-1-carboxaldehyde], **(B)** [2-(2-Aminopropanamido)butanoic acid], **(C)** [Pregan-20-one, 2-hydroxy-5,6-epoxy-15-methyl-], **(D)** [Desogestrel], **(E)** [*2-(2,6-Dimethylphenoxy)-3,6-dimethyl-4- (pentan-3-yloxy)pyridine*], **(F)** [*2,5,8-Trimethyl-1-tetralone*], **(G)** [*1,2,3-Triazole- 4-carboxamide, 5-amino-1-phenylsulfonyl-N-(3-methylphenyl)-*], **(H)** [*Cyclopentanone, 2,5-dicyclopentylidene-*], **(I)** [*1,5- Diphenyl-3-(3-cyclopentylpropyl)pentane*], **(J)** [*8a-Methyl-5-methylene-3-([(pyridin-3-ylmethyl)-amino]-methyl)- decahydro-naphtho[2,3-b]furan-2-one*], **(K)** [*Cidofovir*].

Molecular docking simulations demonstrated that the phytochemical metabolites have strong binding capabilities, forming extensive networks of hydrophobic interactions. These interactions significantly contributed to the predicted stability and binding potential of the compound–protein complexes. Unlike the reference compound, the ten candidate molecules formed more robust and diverse hydrophobic interactions, enhancing their potential as antiviral agents. The interactions between the metabolites and the target residues are illustrated in Figure 10.

#### 3.2.3 Protein molecular dynamics simulation

The RMSF profile obtained from CABSflex2 demonstrated that most residues of the HAdV DNA polymerase protein exhibited low fluctuation values (<2.0 Å), suggesting limited flexibility and a stable overall conformation. Slightly higher fluctuations were observed in loop and terminal regions, which correspond to naturally flexible, solvent-exposed areas of the protein.

**FIGURE 11:**
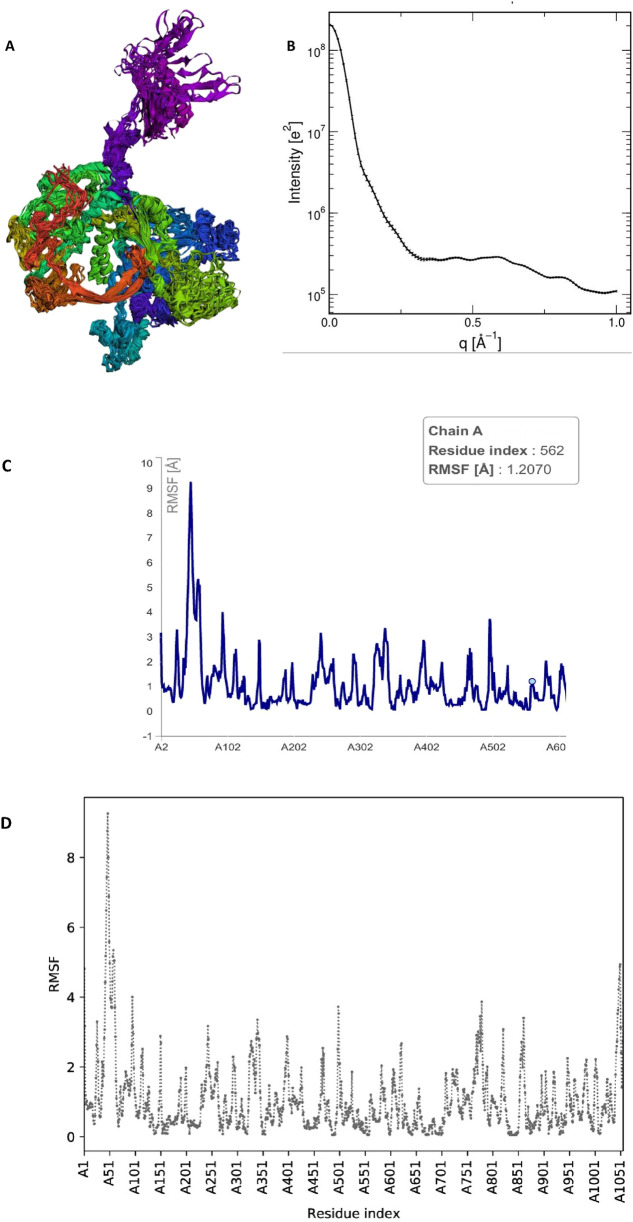
Molecular dynamics (MD) simulation analysis of the HAdV DNA polymerase protein. **(A)** Three-dimensional structure of the protein showing its overall conformation and compact folding during simulation. **(B)** Radius of gyration (Rg) plot showing a stable value (∼37.2 Å), indicating that the protein maintained a compact, folded structure without major expansion or unfolding. **(C)** Root Mean Square Fluctuation (RMSF) profile showing the flexibility of amino acid residues, where only surface loops exhibited minor fluctuations while the core regions remained stable. **(D)** Comparative RMSF plot confirming consistent low residue fluctuations (<3 Å), further supporting the dynamic stability of the protein in the simulation. Collectively, these analyses indicate that the HAdV DNA polymerase retained a stable and well-folded structure under simulated physiological conditions, validating the reliability of the docking results.

The radius of gyration (Rg) calculated from WAXSiS was 37.206 Å, while the solute-only system (without solvent) exhibited an Rg of 36.9079 Å. The small difference (ΔRg ≈ 0.3 Å) indicates that the protein maintained a compact and folded structure throughout the simulation. No significant deviations or unfolding events were detected, confirming the structural stability of the protein during the molecular dynamics simulation Figure 11.

#### 3.2.4 Drug-likeness evaluation

Chemical metabolites and potential therapeutic agents are commonly assessed for drug-likeness using Lipinski’s Rule of Five (Ro5). According to this rule, a compound is more likely to be orally active if it meets the following criteria: a molecular weight (MW) less than 500 g/mol, no more than five hydrogen bond donors (HBDs), no more than ten hydro-gen bond acceptors (HBAs), and a logarithmic partition coefficient (logP) less than 5 (Seliem et al., 2021). Metabolites with low molecular weight are generally more permeable and more readily absorbed, making them favorable candidates for oral administration (Mostafa et al., 2020). In the current study, all ten selected metabolites, in addition to the control compound Cidofovir, were evaluated based on Ro5 parameters. The molecular weights of all tested metabolites were below the 500 g/mol threshold, including CID1 (324.50 g/mol), CID2 (174.20 g/mol), CID3 (345.20 g/mol), CID4 (310.47 g/mol), CID5 (313.43 g/mol), CID6 (188.27 g/mol), CID7 (357.39 g/mol), CID8 (216.32 g/mol), CID9 (334.54 g/mol), CID10 (340.46 g/mol), and the control Cidofovir (279.19 g/mol), as shown in Table 3.

**TABLE 3:** Prediction of the chemical-physical properties.

Compound ID	MW (g/mol)	N or O ≤ 10	NH or OH ≤ 5	MLogP ≤ 5	Violations
CID:1	324.50	0	1	5.05	1
CID:2	174.20	5	3	−0.64	0
CID:3	346.50	3	4	3.49	0
CID:4	310.47	1	1	5.10	1
CID:5	313.43	3	0	3.80	0
CID:6	188.27	0	0	2.85	0
CID:7	357.39	8	2	2.14	0
CID:8	216.32	1	0	3.46	0
CID:9	334.54	0	0	7.45	1
CID:10	340.46	4	1	2.85	0
Cidofovir	279.19	9	4	−2.25	0

Regarding hydrogen bond acceptors, defined as nitrogen and oxygen atoms capable of accepting hydrogen bonds, the metabolites exhibited values ranging from 0 to 8, which is well within the Lipinski limit of fewer than 10; the control compound had an HBA value of 9. Similarly, the number of hydrogen bond donors (HBDs), primarily OH and NH groups, did not exceed the maximum limit of 5 in any of the tested metabolites. The logP values, which reflect compound lipophilicity and influence membrane permeability, were also within the acceptable range for all metabolites and the control, aligning with Lipinski’s criteria. A violation analysis of drug-likeness was also conducted, and the results revealed that several metabolites fully complied with Ro5 with no violations, including CID2, CID3, CID5, CID6, CID7, CID8, and CID10. In contrast, some metabolites exhibited at least one violation, namely CID1, CID4, and CID9. Overall, only 5.2% of the total evaluated parameters represented violations, whereas approximately 60% of the metabolites showed at least one. These findings support the drug-likeness potential of most of the tested *H. speciosa* rhizome metabolites and highlight their suitability as candidates for further drug development. Table 3 summarizes the physicochemical characteristics and compliance of each compound with the Lipinski criteria.

Since all orally administered drug molecules must be water-soluble to ensure effective absorption, three topological prediction models were utilized to evaluate the water solubility of the selected metabolites: ESOL (Daina et al., 2017), Ali (Delaney, 2004), and SILICOS-IT (Ali et al., 2012) methods. According to the ESOL log S model, the predicted log S values for metabolites ranged from −7.47 to 1.67. Among these, three metabolites were classified as moderately soluble, two were highly soluble (along with the control compound), two were poorly soluble, and four were classified as soluble. Using the Ali log S method, solubility values ranged from −9.20 to 2.22. This model similarly indicated that most metabolites fell within the soluble to very soluble or moderately soluble categories, with only three metabolites categorized as poorly soluble. Based on the SILICOS-IT method (Ali et al., 2012), the predicted log S values ranged from −8.92 to 0.17. While this method identified three metabolites as poorly soluble, the remainder demonstrated solubility ranging from high to moderate. Upon evaluating the results from all three predictive models (Table 4), it can be concluded that most of the tested metabolites possess at least moderate water solubility, with several exhibiting good solubility characteristics. These findings suggest that the metabolites are likely to be suitable for both oral and parenteral administration routes.

**TABLE 4:** Water solubility characteristics of *H. speciosa* phytometabolites using three topological methods: ESOL (Daina et al., 2017), Ali (Delaney, 2004), and SILICOS-IT (Ali et al., 2012).

Compound ID	ESOL			Ali			SILICOS- IT		
	Log S	Solubility	Class	Log S	Solubility	Class	Log S	Solubility	Class
CID:1	−5.47	1.11e−03 mg/mL	MS	−6.41	1.28e−04 mg/mL	PS	−4.89	4.21e−03 mg/mL	MS
CID:2	1.67	8.06e+03 mg/mL	HS	2.22	2.91e+04 mg/mL	HS	−0.17	1.18e+02 mg/mL	S
CID:3	−4.26	1.91e−02 mg/mL	MS	−4.45	1.24e−02 mg/mL	MS	−3.57	9.41e−02 mg/mL	S
CID:4	−4.68	6.50e−03 mg/mL	MS	−4.88	4.05e−03 mg/mL	MS	−4.07	2.64e−02 mg/mL	MS
CID:5	−5.57	8.39e−04 mg/mL	PS	−6.47	1.07e−04 mg/mL	PS	−7.19	2.04e−05 mg/mL	PS
CID:6	−3.42	7.22e−02 mg/mL	S	−3.35	8.32e−02 mg/mL	S	−4.33	8.85e−03 mg/mL	MS
CID:7	−3.90	4.51e−02 mg/mL	S	−5.00	3.60e−03 mg/mL	MS	−4.98	3.73e−03 mg/mL	MS
CID:8	−3.24	1.24e−01 mg/mL	S	−3.30	1.08e−01 mg/mL	S	−3.88	2.85e−02 mg/mL	S
CID:9	−7.47	1.14e−05 mg/mL	PS	−9.20	2.10e−07 mg/mL	PS	−8.92	3.99e−07 mg/mL	PS
CID:10	−4.08	2.86e−02 mg/mL	MS	−4.27	1.83e−02 mg/mL	MS	−5.65	7.64e−04 mg/mL	MS
Cidofovir	0.85	1.99e+03 mg/mL	HS	0.88	2.13e+03 mg/mL	HS	0.33	5.97e+02	S

#### 3.2.5 Toxicity risk assessment

Before progressing to clinical trials, *in silico* toxicity assessment represents a critical step in drug development. Computational toxicity prediction has gained increasing importance due to its speed, cost-effectiveness, and ability to reliably evaluate both synthetic and natural metabolites. In this study, the ProTox-II server was employed to evaluate the toxicity profiles and potential adverse effects of the ten selected metabolites. The results, based on the predicted median lethal dose (LD_50_ in mg/kg body weight) and corresponding toxicity classes, are presented in Table 5.

**TABLE 5:** LD_50_ of the *H. speciosa* metabolites and their toxicity class.

Compound	LD_50_ (mg/kg)	Toxicity class	Prediction accuracy (%)
CID1: 5363101	10,000	6	72.9
CID2: 541686	3,000	5	68.07
CID3: 536167	4,290	5	70.97
CID4: 40973	5,000	5	68.07
CID5: 21912293	500	4	67.38
CID6: 255177	570	4	70.97
CID7: 555481	1,190	4	100.0
CID8: 282078	2,920	5	69.26
CID9: 278083	6,430	6	70.97
CID10: 4271074	3,500	5	67.38
Cidofovir: 60613	1,681	4	67.38

Compounds CID5, CID6, and CID7 were classified as toxicity class 4, with LD_50_ values ranging from 500 to 1,190 mg/kg. According to the classification system, these metabolites may pose a risk when administered orally due to their moderate acute toxicity. The control compound, Cidofovir, was also classified as class 4, with an LD_50_ of 1,681 mg/kg.

On the other hand, metabolites CID2, CID3, CID4, CID8, and CID10 were categorized as class 5, with LD_50_ values ranging from 2,920 to 5,000 mg/kg, indicating lower acute toxicity and suggesting suitability for oral or transdermal administration. Notably, CID1 and CID9 exhibited LD_50_ values greater than 5,000 mg/kg and were classified under toxicity class 6, indicating that they are considered practically non-toxic. These findings, summarized in Table 5, highlight the favorable safety profiles of most metabolites and support their potential for further preclinical development.

### 3.3 Vitro studies

#### 3.3.1 Virus titration and median tissue culture infectious dose (TCID_50_)

One of the most critical characteristics of a virus is its ability to spread and replicate efficiently within host cells. To assess viral replication capacity, researchers commonly measure the titer of a specific virus stock, which reflects the number of infectious viral units present in a defined volume. An infectious unit is defined as the minimum quantity of virus capable of producing a detectable response, such as the cytopathic effect (CPE), in cultured cells. A widely used method to quantify infectivity is the median tissue culture infectious dose (TCID_50_), which represents the virus dilution required to infect 50% of a given cell culture. The TCID_50_ provides a standardized measure of viral infectivity and can be calculated using various methods, including the Reed and Muench method, as summarized in Table 6.

**TABLE 6:** Infectivity assay results and TCID50 calculation using the Reed–Muench method.

Dilution	Positive wells/total wells	Cumulative positive (A)	Cumulative negative (B)	Infection rate A/(A+B)	%	PD	TCID 50%
10^−1^	8/8	21	0	1	100	0.09	10^−3.09^
10^−2^	6/8	13	2	0.86	86		
10^−3^	4/8	7	6	0.53	53		
10^−4^	3/8	3	11	0.21	21		
10^−5^	0/8	0	19	0	0		
10^−6^	0/8	0	27	0	0		
10^−7^	0/8	0	35	0	0		
10^−8^	0/8	0	43	0	0		

In this study, the 50% tissue culture infectious dose (TCID50) was calculated as −3.09, indicating that the dilution required to infect 50% of the cell population lies between 10^*−*3^ and 10^*−*4^. This value represents the viral concentration necessary to induce cytopathic effects (CPE) in half of the cultured cells. The infectious dose concentration is the reciprocal of 10^*−*3.09^, providing an accurate quantification of viral infectivity. These results validate the liability of the TCID_50_ calculation in determining the 50% endpoint dilution, as summarized in Table 5.

#### 3.3.2 The cytotoxicity assay (CC_50_) and inhibitory concentration (IC_50_) determination

**FIGURE 12:**
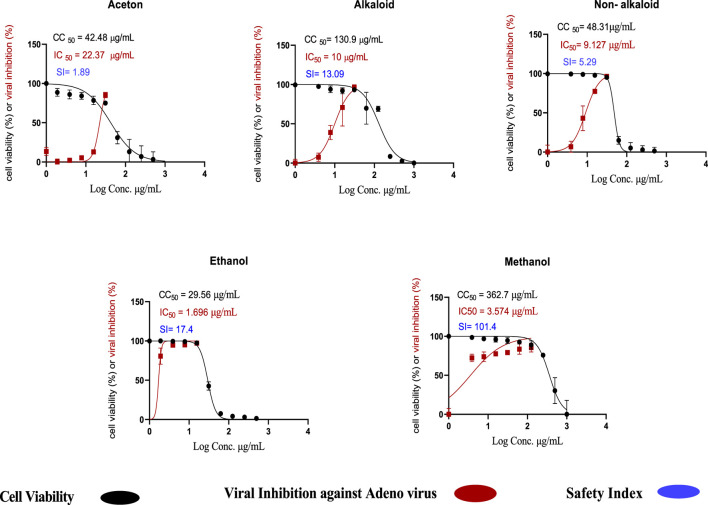
Cytotoxic concentration (CC_50_) and inhibitory concentration (IC_50_) of *Costus speciosus* extracts in Vero cells, determined by nonlinear regression analysis using GraphPad Prism (version 8.0.2). https://www.graphpad.com.

The toxicity of the acetone, methanol, and ethanol extracts of *H. speciosa* rhizome, as well as alkaloid and non-alkaloid fractions, was evaluated using an MTT assay on Vero cells to determine the 50% cytotoxic concentration (CC_50_). Among these, the methanol extracts exhibited the highest CC_50_ value of 362.7 µ/mL, indicating a relatively low cytotoxicity and a higher safety profile in the cell lines tested, as illustrated in Figure 12. The antiviral activity against adenovirus was evaluated using the crystal violet assay. The methanolic extract demonstrated potent pharmacological effects, with an IC_50_ value of 3.57 μg/mL. The ethanol extract also showed notable antiviral activity, with an IC_50_ value of 1.696 μg/mL and a CC_50_ of 29.59 µ/mL, comparatively lower than other extracts Figure 12. It was observed that the percentage of viral growth inhibition increased proportionally with increasing extract concentrations. Figure 12 Concentration–response curves showing cell viability (%) and inhibition of the viral cytopathic effect by the tested *C. speciosus* extracts, generated through nonlinear regression analysis using GraphPad Prism version 8.0.2 (GraphPad Software, San Diego, CA, United States) (Lipinski, 2004). Of particular importance are the selectivity indices (SI), which provide insight into the therapeutic window and safety of each extract. The SI is calculated as the ratio of CC_50_ to IC_50_ and serves as a measure of the balance between cytotoxicity and antiviral activity. Our findings highlight that the methanolic extract possesses a superior safety profile with a high selectivity index (SI = 101.4), indicating potent antiviral activity combined with minimal cytotoxic effects, a desirable attribute in drug development. Table 7 the cytotoxicity, antiviral activity, and selectivity indices for all tested extracts.

**TABLE 7:** Cytotoxic concentration (CC_50_), inhibitory concentration (IC_50_), and selectivity index (SI) of *C. speciosus* extracts.

Extract type	CC_50_ µg/mL	IC_50_ µg/mL	Selectivity index (SI)
Acetone extract	42.48	22.37	1.89
Ethanol extract	29.59	1.696	17.40
Methanol extract	362.70	3.574	101.40
Alkaloid extract	130.90	10.00	13.09
Non-alkaloid extract	48.31	9.127	5.29

#### 3.3.3 Mechanism of viral inhibition

The antiviral assay results demonstrated that *H. speciosa* exhibits significant antiviral activity against human adenovirus type 5 (HAdV-5). Four non-toxic concentrations of the plant extracts (0.5, 1.0, 1.5, and 2.0 μg/mL) were tested across three antiviral mechanisms: adsorption, replication, and virucidal effects. These tests were performed using methanol, ethanol, acetone, alkaloid, and non-alkaloid extracts.

**FIGURE 13:**
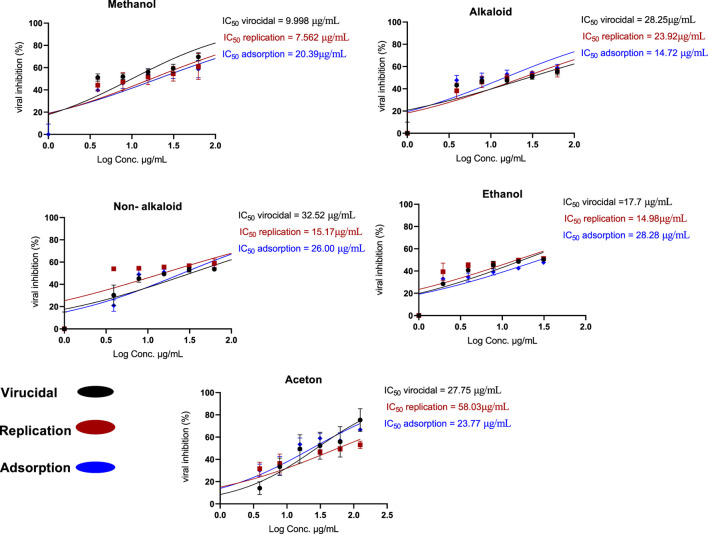
Proposed mechanism of action of *Costus speciosus* extracts against HAdV-5 replication in Vero cells.

The data revealed that the methanol extract of *H. speciosa* effectively inhibited ade-novirus primarily through two dose-dependent mechanisms: viral replication and virucidal activity. As depicted in Figure 13, the suppression of viral replication ranged from 44% to 61%, with an IC_50_ value of 7.562 μg/mL. Virucidal activity inhibition ranged from 51% to 69%, with an IC_50_ of 9.998 μg/mL. In contrast, inhibition of viral adsorption ranged from 39% to 58%, with an IC_50_ of 20.39 μg/mL.

The antiviral effect is attributed to the binding of *H. speciosa* molecules to viral replication proteins, thereby obstructing viral infection by interfering with critical stages of viral replication within infected cells, including blocking the virus’s surface receptors responsible for cell entry.

## 4 Discussion

Viruses are contagious pathogens capable of causing significant harm despite their minuscule size, observable only under a light microscope (Athar et al., 2019). Due to their small size, viruses can infect a wide range of hosts, including humans, and can spread to multiple systems, notably the respiratory system. Respiratory viruses infect cells of the respiratory tract, causing diverse symptoms and contributing significantly to global mortality, ranking among the top ten causes of death worldwide with millions of fatalities annually (Lipinski, 2004). Viral respiratory infections represent a major public health concern, posing serious threats to human health, economic burdens, and challenges especially for vulnerable populations such as children, the elderly, and immunocompromised individuals (Home - GraphPad, 2025).

Human adenoviruses (HAdVs), nonenveloped double-stranded DNA viruses from the Adenoviridae family, include seven species (A–G) from the genus *Mastadenovirus* that infect humans, with over 100 genotypes identified (Gergerich and Dolja, 2006). These viruses cause a variety of clinical conditions, including pneumonia, acute respiratory infections, fever, pharyngitis, and hepatitis, particularly those in group C, leading to increased morbidity and mortality. Adenovirus types 2 and 5 are frequently implicated in respiratory and other infections in children and can cause severe complications in immunocompromised patients. Notably, some children infected with type 5 required liver transplants due to infection severity (WHO, 2025).

Despite advances, effective treatment for adenovirus infections remains challenging due to strain diversity, and no FDA-approved antiviral treatment exists specifically for adenoviruses (Desforges et al., 2019). Although antiviral drugs such as ganciclovir, cidofovir, and ribavirin show varying activity, cidofovir remains the most widely used broad-spectrum antiviral despite concerns about nephrotoxicity (HAdV, 2025). Antiviral agents play a crucial role in controlling viral spread and enhancing host survival, but synthetic drugs may cause side effects. Consequently, traditional remedies and bioactive plant metabolites are increasingly recognized as safer and effective alternatives. Plants have historically provided a rich source of natural products with antiviral properties, including alkaloids, polysaccharides, phytosterols, flavonoids, and carotenoids, many of which have minimal adverse effects when properly used (Gutierrez Sanchez et al., 2022).

*Hellenia speciosa*, also known as “crepe ginger,” is a traditional Indian medicinal plant praised in historical and religious texts. It has demonstrated antiviral properties against influenza A, with reported cytotoxic concentration (CC50) of 117.12 ± 18.31 μg/mL and effective concentration (EC50) of 15.19 ± 0.61 μg/mL in RAW264.7 cells (Waye and Sing, 2010). Additionally, *H. speciosa* extracts have shown activity against vesicular stomatitis virus (VSV) and herpes simplex virus type 1 (HSV-1) (Sagaya Jansi et al., 2021). Recent *in silico* studies further support its antiviral potential, particularly post-COVID-19 pandemic, highlighting high binding affinities to viral targets, though clinical validation is still pending (Dodge et al., 2021).

Despite its medicinal and cultural significance, no prior studies have investigated *H. speciosa* and its active metabolites against human adenoviruses. The present study fills this gap by combining GC–MS-based metabolite profiling, molecular docking, and molecular dynamics (MD) analyses to provide a comprehensive view of the antiviral potential and structural stability of the protein–ligand complexes.

Molecular docking of 112 identified metabolites against the human adenovirus DNA polymerase revealed ten top candidates exhibiting strong binding affinities ranging from –9.1 to –8.7 kcal/mol, significantly surpassing the control drug cidofovir (–5.6 kcal/mol). These compounds formed stable interactions with key catalytic residues including *PHE630, LEU694, ALA810, PHE803,* and *VAL809*, suggesting their potential inhibitory effect on viral replication mechanisms.

To further validate the docking findings, molecular dynamics simulations were performed to evaluate the flexibility and structural stability of the viral DNA polymerase. The RMSF profile obtained from the CABSflex2 server indicated minimal residue fluctuations (below 2.0 Å) across most regions, confirming a stable protein backbone with limited motion in peripheral loops. Additionally, the radius of gyration (Rg) value calculated from the WAXSiS server was 37.206 Å, compared to 36.9079 Å for the solute-only model, demonstrating that the protein maintained a compact and folded conformation without significant structural deviations.

Together, these results suggest that ligand binding does not induce destabilization of the viral polymerase but rather forms stable and energetically favorable complexes, supported by consistent hydrogen bond interactions and limited structural fluctuations. The combined docking and MD findings confirm that the identified metabolites possess strong and stable binding modes, highlighting their potential as novel anti-adenoviral agents targeting the DNA polymerase enzyme.

*In vitro* assays confirmed these findings, with the methanolic extract exhibiting the most potent anti-adenovirus activity against HAdV-5 (IC_50_ = 3.574 μg/mL) and a high selectivity index (SI = 101.4), indicating strong antiviral activity with minimal cytotoxicity. The ethanol extract also demonstrated notable antiviral activity (IC_50_ = 1.696 μg/mL, SI = 17.4). Selectivity indices, which reflect the therapeutic window and safety profile of metabolites, are crucial for drug development, favoring agents that combine high antiviral potency with low cytotoxic effects.

It should be noted that some phytocompounds in *H. speciosa* may belong to the class of Pan-Assay Interference Compounds (PAINS), which are prone to give false-positive results in both *in vitro* and *in silico* assays (Senevirathne et al., 2023). Therefore, all docking and *in vitro* results should be interpreted cautiously. Metabolites identified as top binders were further prioritized based on their chemical properties, plausibility of interactions, and, where possible, literature-reported biological activities. Future studies are required to confirm these findings *in vivo* to validate pharmacological relevance. Moreover, SwissADME predicted that CID4 (Desogestrel) may not be optimal for oral administration due to violations of Lipinski’s rules. However, Desogestrel is an FDA-approved oral contraceptive successfully administered via the oral route (Sharanya et al., 2021). This discrepancy illustrates the limitations of computational ADME predictions and emphasizes the need to integrate *in silico* results with established clinical evidence when assessing drug-likeness.

These results are consistent with previous studies on *H. speciosa* metabolites against other viruses and highlight the potential for further research to validate therapeutic application against adenovirus infections before clinical use. While our results are promising, it should be noted that several plant-derived secondary metabolites are known pan-assay interfering substances (PAINS), which may yield false-positive activity *in vitro* due to nonspecific interactions or assay artifacts (Magalhães et al., 2021). Although our study demonstrated selective antiviral activity and favorable docking interactions for several metabolites, nonspecific effects cannot be fully excluded. Therefore, these results should be regarded as preliminary, and further orthogonal validation, including *in vivo* studies and counter-screening strategies, will be essential to confirm their true pharmacological relevance.

In this study, cidofovir was selected as the reference antiviral agent due to its established mechanism as a DNA polymerase inhibitor and its clinical relevance in adenovirus treatment. Although additional polymerase inhibitors (e.g., Brin cidofovir, ganciclovir, or ribavirin) could have been considered for comparison, these were not included due to practical limitations and variable efficacy against adenoviruses. Future investigations should expand the comparative analysis to include other nucleoside analogues and antiviral agents for a more comprehensive evaluation of therapeutic potential.

## 5 Conclusion

Many viral diseases still lack effective vaccines and antiviral therapies, posing a major challenge to global public health. Natural products represent a valuable reservoir for discovering novel antiviral agents, elucidating new structure–activity relationships, and developing effective prevention and treatment strategies. The present study provides the first evidence that *Hellenia speciosa* (syn. *Costus speciosus*) rhizome extracts exhibit selective activity against human adenoviruses.

Through the integration of GC–MS metabolite profiling, molecular docking, molecular dynamics simulations, and *in vitro* antiviral assays, methanolic extracts were identified as the most potent fraction. Docking and MD analyses revealed that key metabolites formed stable interactions with the viral DNA polymerase, maintaining compact protein conformation (Rg ≈ 37 Å) and low atomic fluctuations, indicating strong structural stability during the simulated complex formation. These computational findings were further supported by experimental results, where the methanol extract demonstrated potent antiviral efficacy (IC_50_ = 3.574 μg/mL, CC_50_ = 362.7 μg/mL, SI = 101.4), particularly during the post-adsorption phase.

However, limitations remain, including the reliance on AutoDock (version 1.5.7) as a docking tool, the absence of metabolite isolation, and the lack of *in vivo* or clinical testing. These factors restrict the immediate translational potential of the findings. Future scope should include animal studies, advanced docking validation with commercial software, and mechanistic assays to confirm mode of action. Additionally, exploring metabolites from other plant parts (bark, roots, flowers) may reveal diverse pharmacological properties. From a scalability perspective, standardized extraction and purification protocols could enable large-scale production, and incorporation into nanoparticle delivery systems may enhance bioavailability. Regarding marketing opportunities, adenovirus infections represent an unmet medical need, particularly in pediatric and immunocompromised populations, where no FDA-approved therapies are currently available. Natural metabolites with high selectivity and favorable safety indices could provide a competitive advantage in the antiviral therapeutics market. In summary, this study highlights *C. speciosus* rhizome as a promising source of anti-adenoviral agents, bridging ethnopharmacology and modern antiviral drug discovery, and offering potential pathways for scalable and marketable biopharmaceutical development.

## Data Availability

The datasets presented in this study can be found in online repositories. The names of the repository/repositories and accession number(s) can be found below: https://pubchem.ncbi.nlm.nih.gov/, 5363101 https://pubchem.ncbi.nlm.nih.gov/, 541686 https://pubchem.ncbi.nlm.nih.gov/, 536167 https://pubchem.ncbi.nlm.nih.gov/, 40973 https://pubchem.ncbi.nlm.nih.gov/, 21912293 https://pubchem.ncbi.nlm.nih.gov/, 255177 https://pubchem.ncbi.nlm.nih.gov/, 555481 https://pubchem.ncbi.nlm.nih.gov/, 282078 https://pubchem.ncbi.nlm.nih.gov/, 278083 https://pubchem.ncbi.nlm.nih.gov/, 4271074 https://pubchem.ncbi.nlm.nih.gov/, 60613 https://www.ncbi.nlm.nih.gov/, P03261.2 https://www.ncbi.nlm.nih.gov/, AAW65499.1 https://www.uniprot.org/, P03261 https://www.rcsb.org/structure/2PYJ, 2PYJ, chain C.
